# Soil pore characteristics and the fate of new switchgrass-derived carbon in switchgrass and prairie bioenergy cropping systems

**DOI:** 10.1038/s41598-024-58444-6

**Published:** 2024-04-03

**Authors:** Kyungmin Kim, Archana Juyal, Alexandra Kravchenko

**Affiliations:** 1 Department of Plant Soil and Microbial Sciences, Michigan State University, East Lansing, MI USA; 2 DOE Great Lakes Bioenergy Research Center, Michigan State University, East Lansing, MI USA; 3 School of Integrative Plant Science, Cornell University, Ithaca, NY USA

**Keywords:** Switchgrass, Prairie, Topography, Carbon sequestration, Soil pore, Plant biodiversity, Plant-derived carbon, X-ray computed micro-tomography, ^13^C pulse labeling, Carbon cycle, Agroecology

## Abstract

Monoculture switchgrass and restored prairie are promising perennial feedstock sources for bioenergy production on the lands unsuitable for conventional agriculture. Such lands often display contrasting topography that influences soil characteristics and interactions between plant growth and soil C gains. This study aimed at elucidating the influences of topography and plant systems on the fate of C originated from switchgrass plants and on its relationships with soil pore characteristics. For that, switchgrass plants were grown in intact soil cores collected from two contrasting topographies, namely steep slopes and topographical depressions, in the fields in multi-year monoculture switchgrass and restored prairie vegetation. The ^13^C pulse labeling allowed tracing the C of switchgrass origin, which X-ray computed micro-tomography enabled in-detail characterization of soil pore structure. In eroded slopes, the differences between the monoculture switchgrass and prairie in terms of total and microbial biomass C were greater than those in topographical depressions. While new switchgrass increased the CO_2_ emission in depressions, it did not significantly affect the CO_2_ emission in slopes. Pores of 18–90 µm Ø facilitated the accumulation of new C in soil, while > 150 µm Ø pores enhanced the mineralization of the new C. These findings suggest that polyculture prairie located in slopes can be particularly beneficial in facilitating soil C accrual and reduce C losses as CO_2_.

## Introduction

Perennial vegetation of North American prairie origin, e.g., monoculture switchgrass (*Panicum virgatum *L.) and restored prairie, are recognized as promising bioenergy cropping systems due to their low maintenance and environmental impacts and potential positive contributions to biodiversity and soil carbon (C) sequestration^1,2^. Compared to annual bioenergy crops, perennial systems can increase soil C storage, due to their longer lifespan, high belowground productivity, and limited soil-disturbance^3–7^. The deep and dense fine root systems of such crops help in allocating high quantities of C underground^8,9^, as well as in increasing plant nutrient and water use efficiency^10^. Perennial bioenergy crops also enhance C protection by facilitating improvements in soil structure through increased production of polysaccharides^9,11,12^. High plant diversity in bioenergy cropping, e.g., as in restored prairie, can be especially effective in promoting larger and faster C sequestration by stimulating microbial activity and generating higher fine root biomass^13–16^.

More importantly, monoculture switchgrass and polyculture prairie vegetation require low nutrient inputs and, thus, are desirable for cultivation on lands otherwise unsuitable for food crop production, which we will refer to here as marginal lands^5,17^. However, most studies of perennial bioenergy crop performances and environmental sustainability are conducted on fertile prime agricultural soils. Unlike prime agricultural soils, marginal lands are often characterized by low fertility and varied, often extreme, topography. Topography is one of the key factors of soil formation that influences, among others, water and soil material redistribution, temperature and humidity gradients, soil organic matter contents, and overall plant growth^18–22^. Topography-induced differences in edaphic and environmental conditions are expected to interact with bioenergy crops’ growth and performance. Nevertheless, how effective will the perennial bioenergy systems be in promoting soil C gains across marginal land terrain with diverse topography is yet to be determined.

Soil pore structure is an important component of soil C gains, since soil pores control fluxes of water and gases, transport of nutrients, and movement of microbes in soil^23–26^. For example, 30–150 µm Ø pores were found to be associated with more intensive processing of plant-derived new C than pores of any other size range^27,28^. Experimental evidence indicates that prevalence of prominent bacterial taxa is associated with pores of this size range, and that they may serve as preferred habitats for certain microbial populations^29,30^. Soil pore size distributions, along with other soil properties, can be affected by both topography^31^ and land use history^32,33^, thus, modifying their influence on C gains and losses.

In a prior study, we used X-ray computed micro-tomography (µCT) to evaluate soil pore characteristics in intact soil cores from multi-year monoculture switchgrass and polyculture restored prairie vegetation (> 18 plant species) from two contrasting topographical positions: eroded slopes and low-lying topographical depressions^34^. We also explored how the new growth of switchgrass plants impacts pore structure and decomposition of existing particulate organic matter (POM). The results demonstrated that higher soil C contents in prairie soils, as opposed to monoculture switchgrass, were associated with larger quantities of 30–90 µm pores and greater solid-pore interfacial areas.

Here, we report on several new results obtained from the experiment described in Juyal et al.^34^. Labeling the growing switchgrass plants with ^13^C enabled detection and quantification of the newly added plant-derived C within the soil, in the microbial biomass, and in the emitted CO_2_. Thus, the focus of the current work is (i) on an in-depth exploration of the fate of new switchgrass-originated C added to the intact soil cores of different vegetation histories and topographies and (ii) on the relationships between the newly added C and soil pore characteristics.

## Materials and methods

### Experimental site and soil sampling

The study site was established in 2010 at Marshall farm of the Great Lakes Bioenergy Research Center at W.K. Kellogg Biological Station, Michigan, USA. Soil samples from two bioenergy cropping systems (i.e., monoculture switchgrass and restored prairie) were collected in the fall of 2018. The vegetation of the restored prairie consisted of 18 species of perennial grasses dominated by *Elymus canadensis*, *Schizachyrium scoparium*, *Sorghastrum nutans*, *Rudbeckia hirta*, and *Rudbeckia triloba.* Two contrasting topographical positions within each system were assessed: (i) low topographical depressions, classified as foot- and toe-slopes (hereafter referred to as “depression”), and (ii) relatively steep and often eroded slopes, classified as shoulders and back-slopes (referred to as “slope”). In each cropping system we randomly selected three pairs of adjacent sampling locations (one from a depression and the other one from an adjacent slope). The layout of the experimental site was provided in Juyal et al.^34^.

From each sampling location we collected two intact soil cores (5 cm Ø, 5 cm h) from 5–10 cm depth (A horizon). The intact cores were carefully sealed on both ends using foil caps, wrapped in aluminum foil, and stored at 4 °C prior to the experiment.

### Switchgrass growth experiment and ^13^C pulse labeling

For the switchgrass growth experiment, half of the cores from each system was planted with switchgrass (referred to as “planted treatment”), while the other half was kept unplanted, (referred to as “unplanted control”). One of the two cores from each sampling location was randomly selected for one of the two treatments. Two–three switchgrass (var. Cave-In-Rock) seeds were placed in each core of the planted treatment. The seeds were covered with a thin layer (~ 1 mm) of garden mix soil to enhance germination. After the seedlings established, only one plant was kept per soil core. Both plant and control cores were kept in the greenhouse and watered daily to maintain constant moisture content of 45–50% water-filled pore space (WFPS), with daily water losses determined by weighing the cores. Average temperatures of 25 °C during the day and 22 °C at night were maintained in the greenhouse.

**Figure 1:**
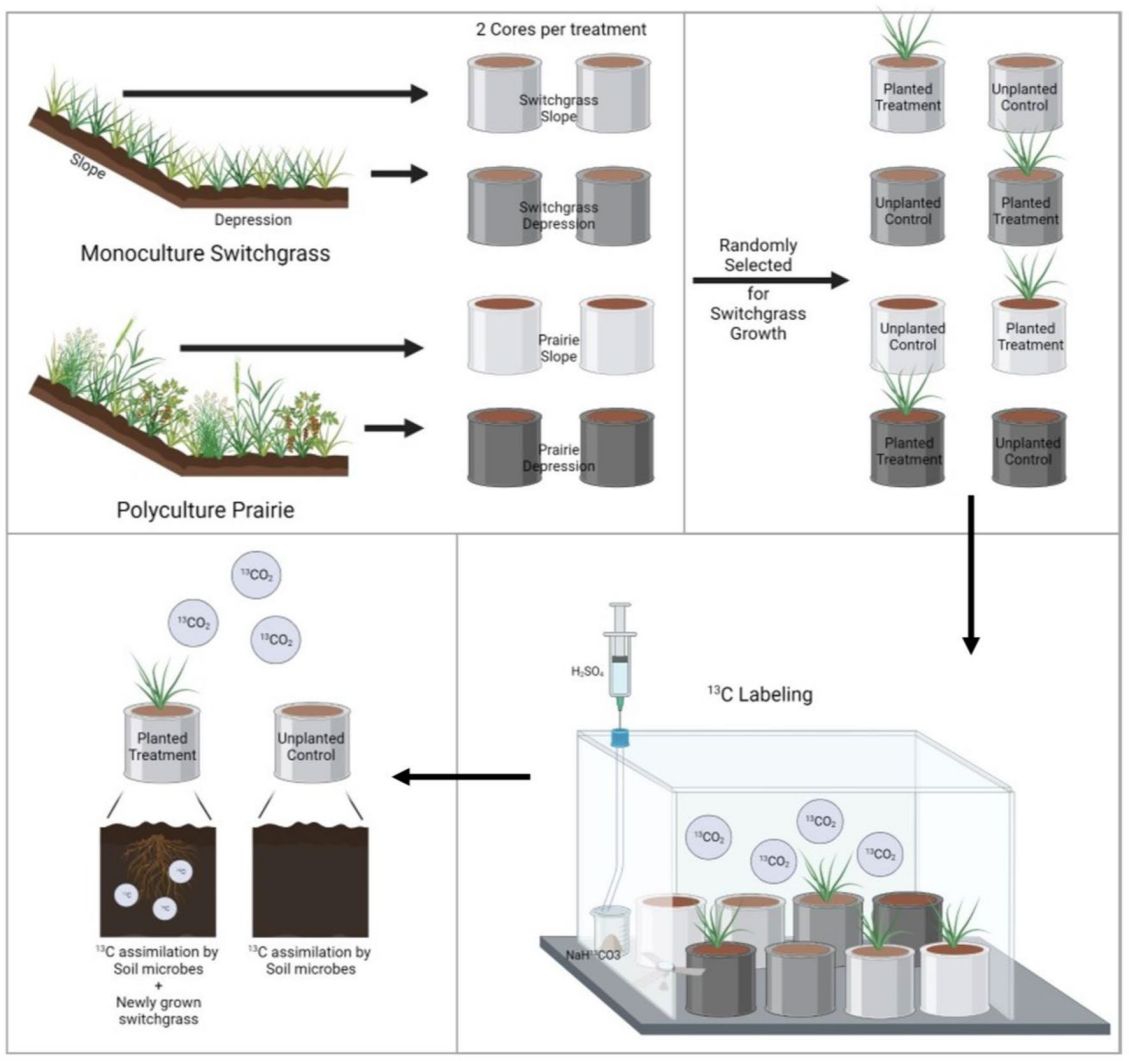
Pipeline diagram for soil sampling, switchgrass growth, and labelling procedure (created in Biorender.com).

The ^13^C pulse labeling of the switchgrass plants started 30 days after germination. The plants were pulse-labeled for 3 weeks, with one 6-h pulse per week. The labeling was conducted by placing the cores in an air-tight glass chamber (80 × 60 × 60 cm). The chamber was sealed from an outside atmosphere by placing it into a water-filled tray. A glass beaker with 110 mg of 98% ^13^C enriched NaH^13^CO_3_ was placed inside the chamber and connected via plastic tubing to a syringe. 1 M H_2_SO_4_ was slowly injected through the syringe in excess to react with all NaH^13^CO_3_, leading to ~ 40 atom% of ^13^CO_2_ in the chamber’s headspace^35^. A small electric fan was placed next to the beaker to ensure circulation of ^13^CO_2_ within the chamber. During labeling the temperature within the chamber was monitored using an installed thermometer and was maintained between 25 and 30 °C. Note that control (unplanted) cores were subjected to labeling together with the planted cores. Pipeline diagram for soil sampling, switchgrass growth, and labeling was provided in Fig. 1.

After labeling, the cores were placed in a ventilated area under daylight lamps to further promote photosynthetic activity. After the last labeling pulse, the plants were left to grow for another week and harvested afterward. After the harvest, the aboveground plant biomass was dried at 30 °C for weight determination. The dried plants were cut and ground into fine powder using mortar and pestle for ^13^C and total C analyses.

### Soil pore characterization

Soil pore characterization was performed as described in Juyal et al.^34^. Briefly, soil cores were scanned after the plant harvest using an X-ray computed tomography (μCT) system (North Star Imaging, X3000, Rogers, USA) in the Department of Horticulture at Michigan State University. The soil cores were scanned with energy settings of 75 kV and 450 μA with 2880 projections. The X-ray μCT images had a resolution of 18.2 μm, and were reconstructed using efX software (North Star, Rogers, USA). Image analyses for soil pore characterization were performed in ImageJ software^36^. Prior to main image analysis, the X-ray μCT images were subjected to background removal using Xlib plugin^37^, to eliminate random noise and scanning artifacts. The preprocessed images were then segmented into solids and pores using adaptive window indicator kriging method^38^. From the segmented images, soil pore characteristics, including visible porosity, pore connectivity, and solid-pore interface was determined using the 3D Minkowski functionals^38^. We define visible porosity as pores that can be identified on the soil images scanned at 18.2 µm resolution, that is the pores > 18.2 µm Ø. We define pore connectivity as the fraction of the pore volume connected to the external surface of the core, and solid-pore interfacial area as the number of solid voxels directly bordering the pore space of the core. Pore-size distribution was determined using Xlib plugin^37^. We combined the studied pores into the following size classes 18–30, 30–90, 90–150, 150–250, and 250–350 µm Ø. Pore size classes was set based on the literature review: the pores of 6–40 µm Ø are associated with carbon accrual^39^, while the new source of C pores is preferentially added to pores of 15–90 µm Ø^40^. Pores of 60–180 µm Ø are highly related to microbial activities^41^, and pores of < 300 µm Ø are the routes for water and nutrient supplies to microorganisms^42^. Thus, our size class 18–30 µm Ø was expected to be the place for old carbon storage, 30–90 µm Ø for the main place for new C accrual, 90–150 µm Ø for microbial processing of organic C, and 150–350 µm Ø for the pathways of water and nutrients.

### Soil core incubation and analysis

After μCT scanning, the soil cores were subjected to a 10-day incubation. The cores were placed into Mason jars, containing small beakers with water to minimize evaporation, and kept at ~ 22 °C in the dark. Gas samples were collected from the jars on day 1, 3, 5, 7, and 10 for ^13^CO_2_ and total CO_2_ analyses. At each collection event, luer-lock syringes with needles were injected to Mason jar and the suction-injection was repeated 3 times to mix the headspace homogeneously. Then 20 mL of gas extracted from the Mason jar was stored in the 20 mL glass vials sealed with rubber septa until the gas analysis. The CO_2_ concentration was analyzed using Licor infrared gas analyzer, and the isotopic composition of the CO_2_ was measured using Isoprime 100 IRMS located at Michigan State University. Mason jars were ventilated to the room air after each gas collection event to avoid the saturation.

After the last gas sampling, the cores were dismantled, and roots were separated from the soil. In preparation for further analyses, the soil was cleaned from visible root residues, air-dried, and ground into fine powder. The roots were carefully brushed to remove any attached soil pieces, air-dried, and ground.

Soil microbial biomass was determined using modified chloroform fumigation-extraction method^43^. For each soil core, a 5 g of soil was fumigated with chloroform for 24 h, while another 5 g of soil was left non-fumigated. Both fumigated and non-fumigated samples were shaken with 25 mL of 0.05 M K_2_SO_4_ solution for 15 min, centrifuged at 3000 rpm for 10 min, and filtered (Whatman no.3). The K_2_SO_4_ extracts were freeze-dried for ^13^C and total C analyses. Microbial biomass C was calculated as the difference in dissolved organic C contents between fumigated and non-fumigated soil extracts. A conversion coefficient (K_EC_) value of 0.45 was used to estimate the microbial biomass C^44^.

For ^13^C analysis of plant and soil samples and in K_2_SO_4_ soil extracts, 2–3 laboratory replicates of each sample were weighed and packed in tin foil containers. The δ^13^C in the samples was measured using an elemental analyzer (Vario ISOTOPE CUBE, Elementar) coupled to an isotope ratio mass spectrometer (Isoprime Vision, Elementar). The ^13^C enrichment was reported as δ^13^C (‰) based on the PeeDee Belemnite standard, and converted to Atom%^13^C using the following equation:

$${\text{Atom}}\%^{13} {\text{C}} = \{ [(\delta^{13} {\text{C}} \cdot 1000^{ - 1} + 1) \cdot 0.0112375]^{ - 1} + 1\}^{ - 1} \cdot 100$$

where 0.0112375 represents the ratio of ^13^C to ^12^C.

Then the enrichment of ^13^C in excess of the natural abundance (Atom%^13^C_excess_) was calculated by using the following equation:

$${\text{Atom}}\%^{{{13}}} {\text{C}}_{{{\text{excess}}}} = {\text{Atom}}\%^{{{13}}} {\text{C}}_{{{\text{labeled}}}} - {\text{Atom}}\%^{{{13}}} {\text{C}}_{{{\text{non}} - {\text{labeled}}}}$$

where Atom%^13^C_labeled_ indicates atom%^13^C of the labeled plant or soil materials, and Atom%^13^C_non-labeled_ indicates atom%^13^C of the non-labeled plant or soil materials.

### Statistical analysis

A mixed-model approach was used for comparisons between the two studied bioenergy cropping systems and between the two topographical positions using PROC MIXED procedure of SAS version 9.4 (SAS Institute Inc., NC, USA). The statistical model used to assess the effects of cropping systems and topography on C and plant-derived C (% C and atom%^13^C excess) consisted of fixed effects of tissue (shoot and root), systems (prairie and switchgrass), topography (slope and depression) and their interactions. Soil cores were added to the model as a random factor nested within the system and topography and were used as an error term for testing the system and topography effects. The statistical model for soil C, microbial biomass C, and cumulative CO_2_-C from 10-day mineralization experiment consisted of systems, topography, planted treatment, and their interactions as fixed effects. These models also included the random effect of individual soil cores nested within systems, topography, and planted treatment. The statistical model for dynamics of ^13^C-CO_2_ during mineralization consisted of the fixed effects of systems, topography, planted treatment, day of incubation, and their interactions. Repeated measures approach was implemented as described in Milliken and Johnson^45^, with the soil cores as the subject of repeated measurements. The variance–covariance structure was determined by evaluating AIC and BIC values.

The associations between three soil pore metrics obtained from image analysis, namely, pore connectivity, solid-pore interface, and volumes of pores of the studied size classes, and newly added C and soil pore characteristics were examined using regression analysis via PROC REG procedure of SAS. In order to ensure that data from both cropping systems can be combined in the regression analyses, we had to account for the differences in the quantities of new C being added into the system. Thus, soil ^13^C and microbial biomass ^13^C were normalized by reporting them on per g ^13^C of aboveground biomass basis. The ^13^C of the aboveground biomass was selected as opposed to that of the belowground biomass as an indicator of the new C additions, because of greater uncertainty and lower accuracy in belowground ^13^C measurements. The regressions were initially conducted separately for prairie and monoculture switchgrass systems, and when the slopes of the two system were not significantly different from each other, the two groups were combined, and a single regression analysis performed. Since the goal of analyzing the relationships between pore size groups and newly added C was to decipher the general contributions of pores of different sizes to the fate of new C, the analyses were conducted across all systems and topographical positions.

The normality assumption was assessed by visual inspection of normal probability plots, and the homogeneity of variance assumption was examined using Levene's test^45^. In cases where normality assumption was not met (e.g., soil C and atom%^13^C of microbial biomass), the variables were natural log transformed. When the equal variance assumption was violated, the model for unequal variance was used^45^. The interaction effects were examined using slicing (i.e., F-tests for simple effects), with further mean separations using t-tests conducted when slice F-tests results were statistically significant at 0.10 level. The statistical tests were reported as significant when the p-values were less than 0.01 (***), 0.05 (**), and 0.10 (*).

## Results

### Biomass, total C, and ^13^C of newly grown switchgrass plants

**Figure 2:**
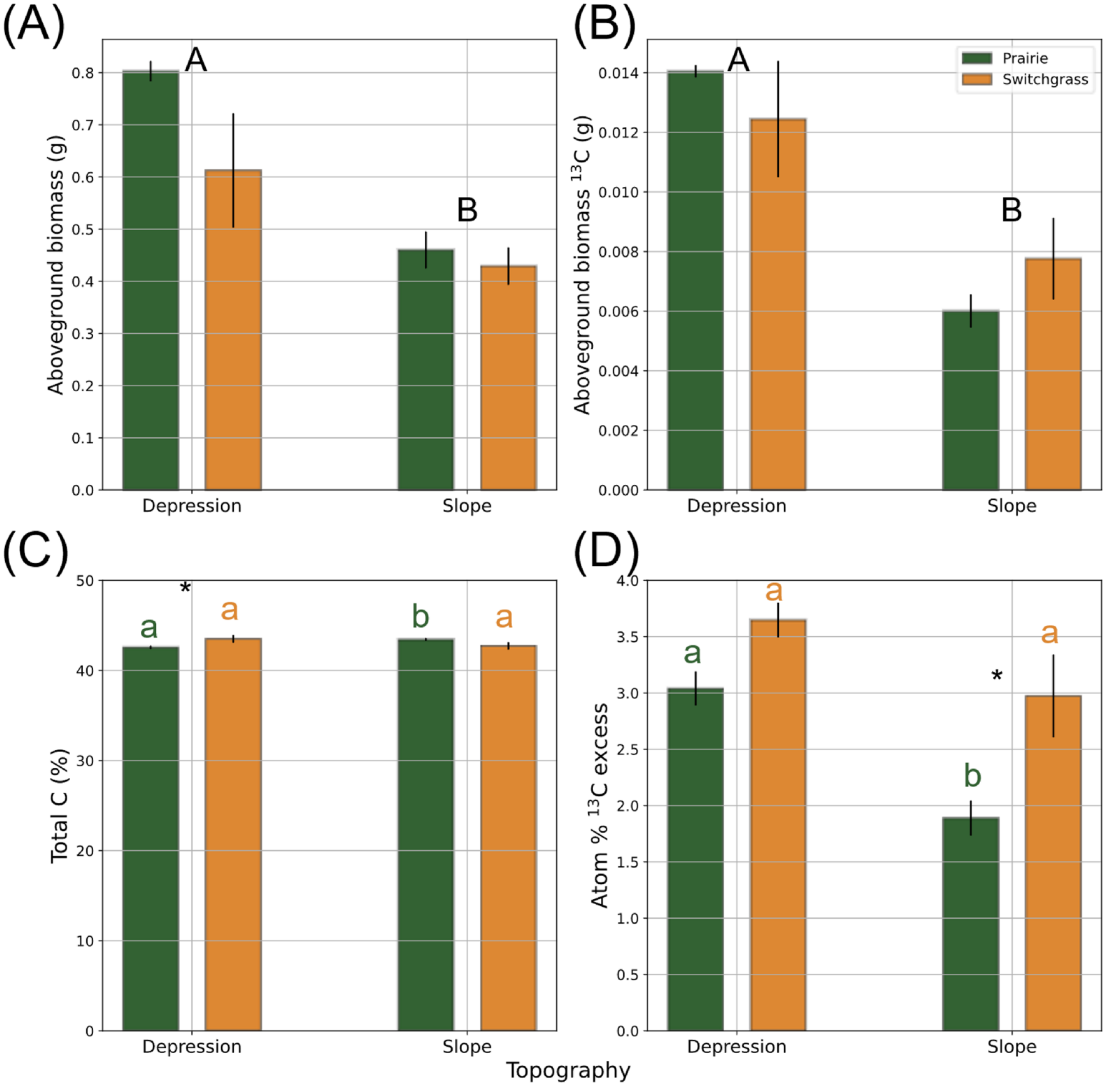
Aboveground biomass (**A**), aboveground biomass ^13^C (**B**), aboveground % C (**C**), and aboveground atom %^13^C excess (**D**) of switchgrass grown in the soils from multi-year monoculture switchgrass and restored prairie cropping systems in topographical slopes and depressions. Shown are means with error bars representing standard errors. Capital letters mark significant differences between topographical positions (p < 0.05). Lowercase letters mark significant differences between topographical positions within the same system and biomass type. Asterisk * indicates the significant difference between the two system (prairie vs. switchgrass) within the same topography and biomass type (p < 0.10).

The aboveground biomass of switchgrass plants grown in depression soils was significantly higher than in slope soils (p < 0.05, Fig. 2A), and numerically higher in prairie than in switchgrass soil (p = 0.45). The aboveground biomass of the switchgrass plants grown in soils from both prairie and switchgrass systems had higher C content compared to the belowground biomass (p < 0.01, Table S1). Total C in the aboveground biomass was higher in the switchgrass system compared to the prairie system (Fig. 2C), while the belowground biomass C was higher in the prairie system rather than the switchgrass system (Table S1). In the prairie system, both above- and belowground switchgrass biomass C were greater in soil from the slopes than in the depression soil (Fig. 2C and Fig. S1A).

Total ^13^C and the atom% ^13^C excess in the switchgrass aboveground biomass tended to be higher when grown in depression rather than in slope soils (p < 0.10, Fig. 2B,D and Table S1). Atom% ^13^C excess of the belowground biomass was the greatest in prairie depression soil, and the lowest in prairie slope soil (Fig. S1B).

### Soil C and microbial biomass C

**Figure 3:**
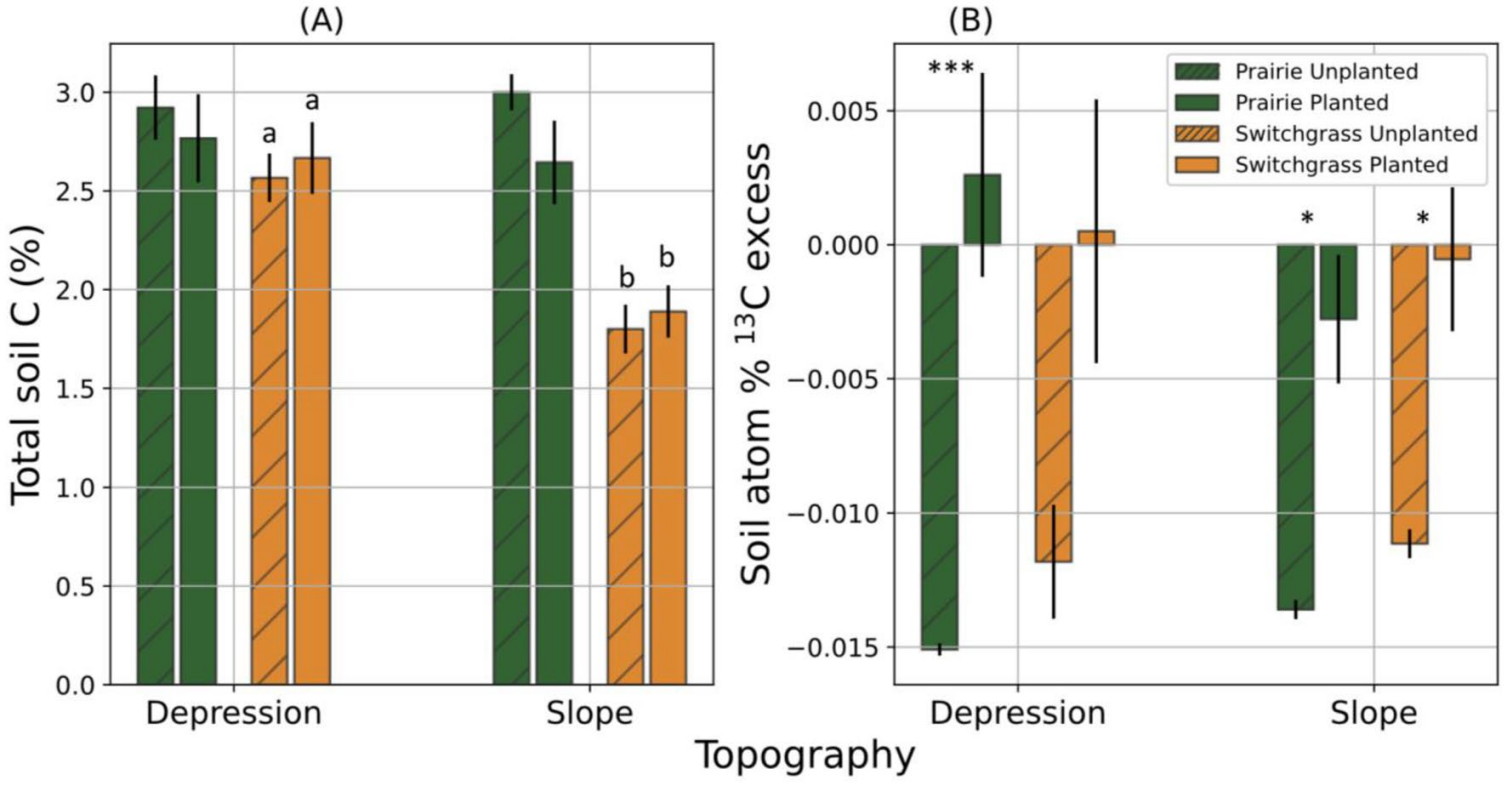
Total C (**A**) and atom%^13^C excess (**B**) of soils from multi-year monoculture switchgrass and restored prairie cropping systems in topographical slopes and depressions. Shown are means with error bars representing standard errors. Bars with and without hatched pattern represent unplanted controls and switchgrass planted (+ Plant) treatments. Asterisks *** and * indicate that the differences between plant treatments (unplanted controls vs. + Plant) is significantly different from 0 (p < 0.01 and p < 0.10, respectively). Letters mark significant differences between topographical positions within the same system and plant treatment.

**Figure 4:**
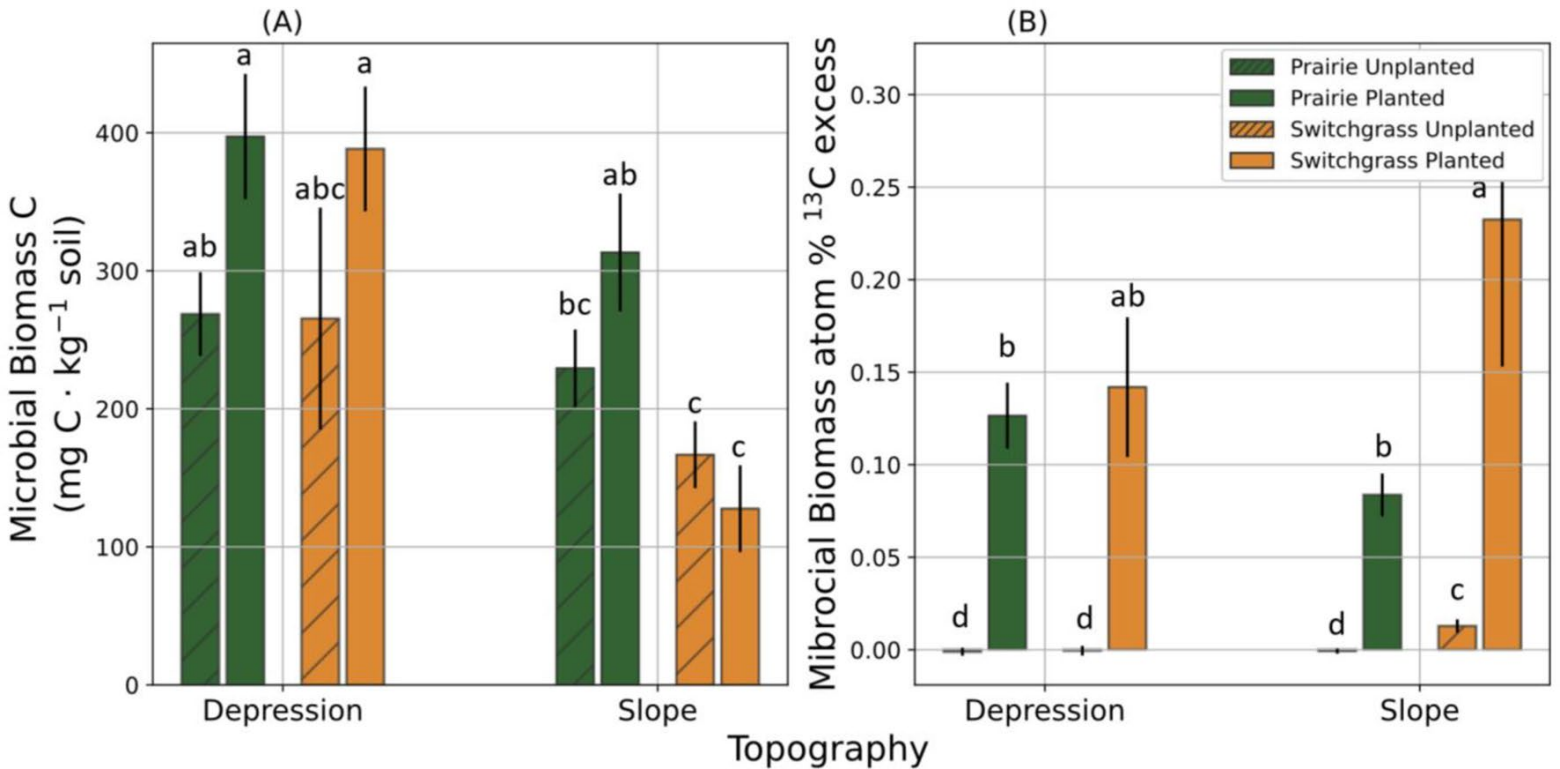
Microbial biomass C (**A**) and microbial biomass atom%^13^C excess (**B**) in soils from multi-year monoculture switchgrass and restored prairie cropping systems in topographical slopes and depressions. Different letters indicate the difference between treatments according to the all-means comparison result (p < 0.10). Data shown are means with error bars representing standard errors.

Planted treatment did not significantly change the total C in the soil (Table S2). Total soil C content in unplanted control was greater in prairie soils than in switchgrass soils (p < 0.01), and greater in depression than in slope soils (p < 0.10, Fig. 3A and Table S2). Planted treatment had a similar trend, but the difference between cropping systems was only significant in slope soils (Fig. 3A). Total microbial biomass C was greater in depression than in slope soils (p < 0.01, Fig. 4A and Table S2), while the difference between prairie and switchgrass soils was not statistically significant. Across all studied systems and topographical positions, the microbial biomass C of planted treatments was greater than that in the unplanted controls (F-test, p < 0.10, Table S2). However, that trend was not present in the switchgrass soil from slopes (Fig. 4A).

Atom% ^13^C excess of prairie soil significantly increased when the new switchgrass was grown in both topographical positions (Fig. 3B). Atom% ^13^C excess of switchgrass soil significantly increased in slope and numerically only in depression soils. There were no significant effects of either the system or topography in planted treatments. Microbial biomass in the planted treatment was significantly enriched in soils of both systems and topographical positions when compared to unplanted controls (p < 0.01, Fig. 4B), and enrichment was stronger in the switchgrass than in the prairie system (p < 0.05), particularly strong in the slope soil of the switchgrass (Fig. 4B).

### CO_2_ mineralization

**Figure 5:**
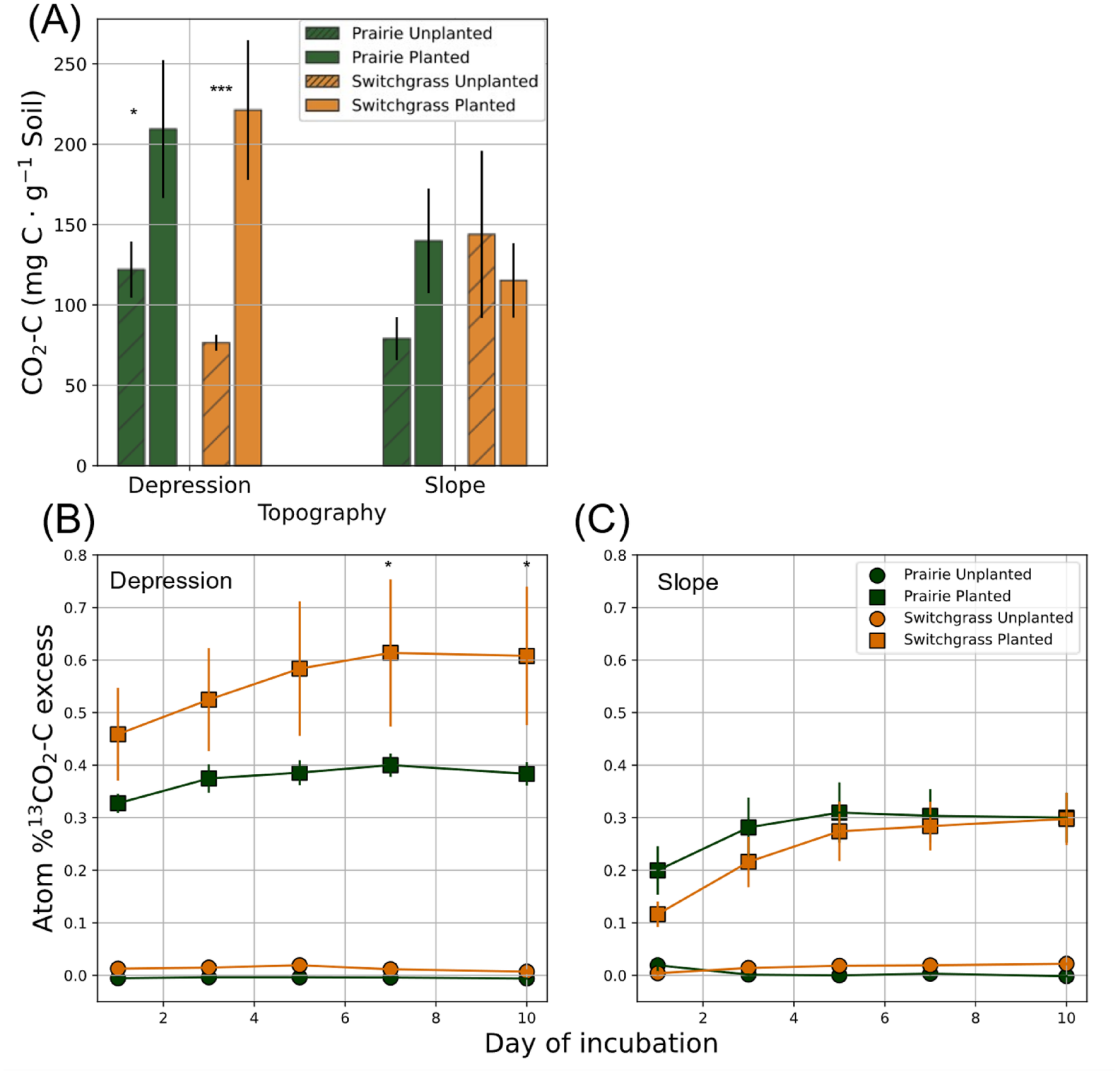
CO_2_ emission from soils of contrasting systems and topography (10-day mineralization test). Cumulative CO_2_ emission from soil cores (**A**), and atom % ^13^C excess of the emitted CO_2_ during the incubation in depression soils (**B**) and slope soils (**C**) are presented. Asterisk * mark the significant differences between the system under given plant treatment and system (p < 0.10). Data shown are means with error bars representing standard errors.

The cumulative CO_2_ emitted from the planted treatment was significantly higher than that from unplanted controls in both switchgrass and prairie soils of depression (p < 0.10 and 0.01, Fig. 5A). On the contrary, there was no difference between the planted and unplanted treatments in the slope. At both topographical positions, the atom %^13^CO_2_-C excess was higher in planted cores than in unplanted controls (p < 0.01, Fig. 5B,C, and Table S3). Atom%^13^C of the CO_2_ emitted during the incubation was higher in the depression than slope soils, across both systems (Fig. 5B,C). It was particularly high in the switchgrass soil of depression and became significantly higher than that of the prairie at the end of the incubation (8–10 days) (Fig. 5B).

### Effect of soil pore characteristics on newly added plant ^13^C

**Figure 6:**
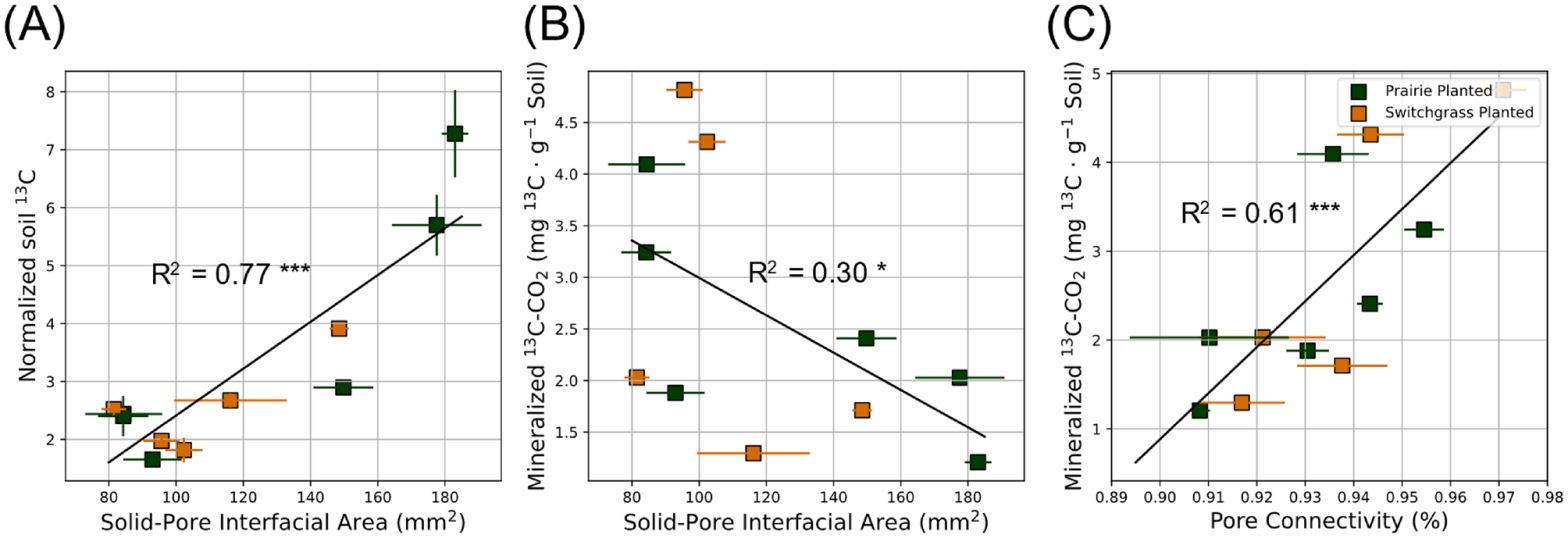
Relationship between ^13^C (new C of switchgrass origin) and pore characteristics in the two contrasting bioenergy system (prairie and switchgrass) after 3 months of new switchgrass growth. Shown are relationships between solid-pore interfacial area and soil ^13^C (**A**), solid-pore interfacial area and mineralized ^13^C (**B**), and pore connectivity and mineralized ^13^C (**C**). Soil ^13^C was normalized by accounting for ^13^C in the aboveground biomass. Solid lines represent regression models fitted for the planted treatments (Prairie and Switchgrass), with R^2^ values and significance level (*** for p < 0.01 and * for p < 0.10). Error bars represent standard errors.

**Figure 7:**
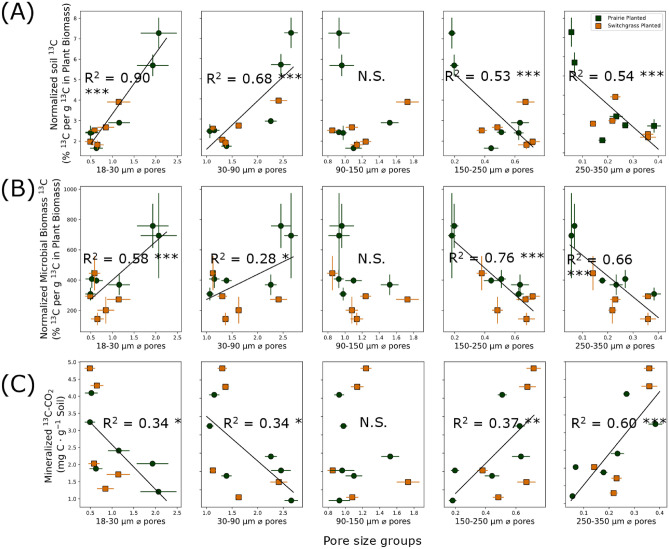
Relationship between soil pore volume of different pore size groups and soil ^13^C (**A**), relationship between soil volume of different pore size groups and microbial biomass ^13^C (**B**), and relationship between soil volume of different pore size groups and mineralized ^13^C (**C**). Pore size groups are 0–30, 30–90, 90–150, 150–250, and 250–350 µm Ø (from left to right). Soil ^13^C and microbial biomass ^13^C was normalized by dividing % ^13^C in soil by g ^13^C in aboveground biomass to exclude the effect of C increase directly from plant biomass. Mineralized ^13^CO_2_ is the result from the 10-day incubation experiment after the newly grown switchgrass is terminated. Solid lines represent regression models fitted for the planted treatments (Prairie and Switchgrass), with R^2^ values and significance level (*** for p < 0.01 and * for p < 0.10). Error bars represent standard errors.

Solid-pore interfacial area was positively related to soil ^13^C (Fig. 6A). High pore connectivity and smaller solid-pore interfacial area led to greater mineralized ^13^C (Fig. 6B,C). Volumes of soil pores in 18–30 µm and 30–90 µm Ø size ranges were positively related to the newly added soil ^13^C and microbial biomass ^13^C (Fig. 7A,B). In 90–150 µm Ø pore size range, the pore volumes did not significantly affect the fate of newly added plant ^13^C. The volumes of larger pores (> 150 µm Ø) were negatively associated with soil ^13^C and microbial biomass ^13^C. The associations between pore volumes and mineralized ^13^CO_2_-C were opposite of those of soil and microbial biomass ^13^C. That is, volumes of 18–90 µm Ø pores were negatively related to mineralized ^13^C, while its relationship with > 150 µm Ø pores was positive (Fig. 7C).

## Discussion

### Switchgrass performance

As expected, switchgrass grew better in more fertile depression soils than in the soil of the slopes (Fig. 2); the result is consistent with numerous previous studies where higher biomass and yield of switchgrass, corn, and winter wheat, among many other crops, were observed in toe-slope rather than shoulder topographic positions (e.g., ^46–48^). Interestingly, the young switchgrass had a greater atom %^13^C excess of its aboveground biomass when it was grown in the soil of monoculture switchgrass history rather than that of prairie (Fig. 2D). The similar numeric trend was also present in the belowground biomass data on slopes (Fig. S1B). Similar to other crops, new switchgrass prefers to utilize existing biopores generated by other switchgrass plants, which allows lower resistance to root growth^49–51^. In contrast, when grown in prairie soil, the new switchgrass relies more on soil matrix pores, leading to a delayed root growth, but greater exploration of the entire soil matrix^50^. We attribute greater belowground biomass in the monoculture switchgrass system of this study in part to the availability of existing biopores, which facilitated quick root growth of the young plants.

### Newly added ^13^C in soil, microbial biomass, and CO_2_ emissions

Topographical influences on soil organic and microbial biomass C, i.e., higher values in depression than in slope soils (Figs. 3A and 4A), were expected and consistent with previous studies^20,22,52–54^, reflecting more fertile, nutrient rich, and moisture available environments of topographical depressions. Footslopes also have greater total microbial, bacterial, and arbuscular mycorrhizal fungal biomass than shoulder positions^54^.

Greater C in the soils of prairie as compared to that of the switchgrass system (Fig. 3A and Table S2) was in line with our previous study^34^ as well as other reports of soil C accrual stimulation by multiple years of biodiverse perennial vegetation^55,56^. But the soil C gain benefits of prairie over monoculture switchgrass vegetation appeared to vary across topographically diverse terrain. The differences in C contents and in microbial biomass C between polyculture prairie and monoculture switchgrass systems were more pronounced in the soils of topographical slopes than in depressions (Figs. 3A and 4A). These findings suggest that polyculture bioenergy systems may be particularly beneficial when placed on eroded slopes of marginal land terrain^57^.

The highest enrichment of microbial biomass by C from the new switchgrass growth was observed in the soil of switchgrass system on slopes (Fig. 4B), suggesting that the resident microbial community in this soil was better able to utilize the newly added C than that in the prairie soils and in both prairie and switchgrass soils of topographical depressions. It is possible that the microbial communities of monoculture switchgrass system on eroded low fertility slopes became adapted to low C environment, developing an ability to more efficiently assimilate new C source into their biomass^58–60^. Yet, despite such apparently more efficient utilization of newly added C inputs in the soil from slopes with switchgrass history, the overall microbial biomass C was still much greater in fertile depression and prairie soils.

Higher microbial biomass C in the soil subjected to new switchgrass growth as compared to that in unplanted controls (Fig. 4A) was an expected outcome of the positive effect of live plants and their root exudates on soil microorganisms^61,62^. New plant growth increased mineralized CO_2_-C emissions of both plant systems in depression, but not in slope positions (Fig. 5A), and newly added C from labeled switchgrass plants apparently was the main contribution to such an increase (Fig. 4B). The lack of new plant growth effect on total CO_2_ emissions was especially strong in the soil of switchgrass system on slope (Fig. 5A), and atom%^13^C excess of the emitted CO_2_ was the lowest there as well (Fig. 5C). Together with microbial biomass results (Fig. 4B), these observations support the notion that the microbial communities there were minimizing losses of newly added C, assimilating it into microbial biomass instead^58–60^, suggesting their potentially higher C use efficiency. Low nutrient contents and a stressful environment of slope soil may have facilitated greater microbial assimilation and less microbial respiration of newly added C^63^. We surmise that, on the contrary, the microbial community in depression soils was used to nutrient availability, leading to high respiration rates in the presence of newly added C^60^.

### Effect of soil pore characteristics on newly added plant C

Our results emphasized the importance of soil physical characteristics for newly added plant-derived C in soil, microbial biomass, and as emitted CO_2_. The size of solid-pore interfacial area positively influenced the amount of newly added ^13^C that remained in the soil after the incubation and decreased its losses as CO_2_ (Fig. 6A,B). The size of the interface between soil solid materials and pore space is what defines the potential for microbial degradation byproducts and microbial necromass to engage with soil minerals and gain protection through physico-chemical bonding^28,34,64^. By utilizing ^13^C pulse-labeling, our study provided additional evidence that an increased solid-pore interface can promote the accumulation of recently incorporated C (Fig. 6A) while concurrently minimizing the loss of the new C (Fig. 6C). On the other hand, high pore connectivity can promote the diffusion of gas produced from organic sources, thereby increasing CO_2_ mineralization (Fig. 6C).

Our study underscores the varying impact of pores of different size groups on the balance between C accumulation and loss. While volumes of 18–90 µm Ø pores were positively associated with the new C both in the soil and in the microbial biomass, the trends were negative for > 150 µm Ø pores (Fig. 6A,B). On the contrary, the losses of ^13^CO_2_ were negatively related to 18–90 µm Ø and positively to > 150 µm Ø pores (Fig. 7C). The results suggest that 18–90 µm Ø pores were associated with accumulation of newly added C in soil and microbial biomass, while the large pores facilitated decomposition and CO_2_ emissions. It is consistent with previous studies that reported that < 40 µm Ø pores were associated with C protection^27^, and those with 30–150 µm Ø play a crucial role in C gains^28,34^. Microbial enzyme activities are positively associated with pores of 60–180 µm Ø ^41^, which may have led to greater assimilation of recently added C into microbial biomass at similar or smaller (18–90 µm Ø) pore size ranges (Fig. 7B). Our results provide further evidence that such pore size ranges function as optimal microbial habitats and are linked to higher microbial abundance^24,29^.

## Conclusion

Our study provides evidence that polyculture bioenergy systems can be particularly beneficial when placed on eroded slopes of marginal land terrain. There were more pronounced differences in C contents and in microbial biomass C between polyculture prairie and monoculture switchgrass systems in the soils of topographical slopes when compared to slopes. It suggests that additional accumulation of C can be better achieved by the land conversion from monoculture to polyculture. A lack of new plant growth effect on total CO_2_ emissions in slopes reinforces the advantages of employing polyculture systems in such topography. It is likely attributed due to higher C use efficiency of the microbial communities in slopes as a result of adaptation to low-nutrient environments. Our study also underscores the varying impact of pore characteristics on the balance between accumulation and losses of new plant-derived C. An increased solid-pore interface can promote the accumulation of recently incorporated C, while concurrently minimizing the loss of the new C. On the other hand, high pore connectivity can promote the diffusion of gas produced from organic sources, thereby increasing CO_2_ mineralization. Pores of 18–90 µm Ø can facilitate the accumulation of new C in soil, while > 150 µm Ø pores can enhance the mineralization of the new C. Building upon our study’s findings, a valuable next step could involve investigating mechanisms underlying carbon accrual in different topography and bioenergy systems, focusing on the interplay between soil pore structure and microbial communities.

### Supplementary Information

Supplementary Information.

## Data Availability

The datasets used and/or analyzed during the current study available from the corresponding author on reasonable request.
