# Supplementary material for: Soil pore characteristics and the fate of new switchgrass-derived carbon in switchgrass and prairie bioenergy cropping systems
Source: Sci Rep. 2024 Apr 3;14:7824. doi: 10.1038/s41598-024-58444-6 (PMC10991283; doi:10.1038/s41598-024-58444-6)
Supplement: Supplementary file 1 — Supplementary Information. [file 41598_2024_58444_MOESM1_ESM.docx]

# Supplementary Materials


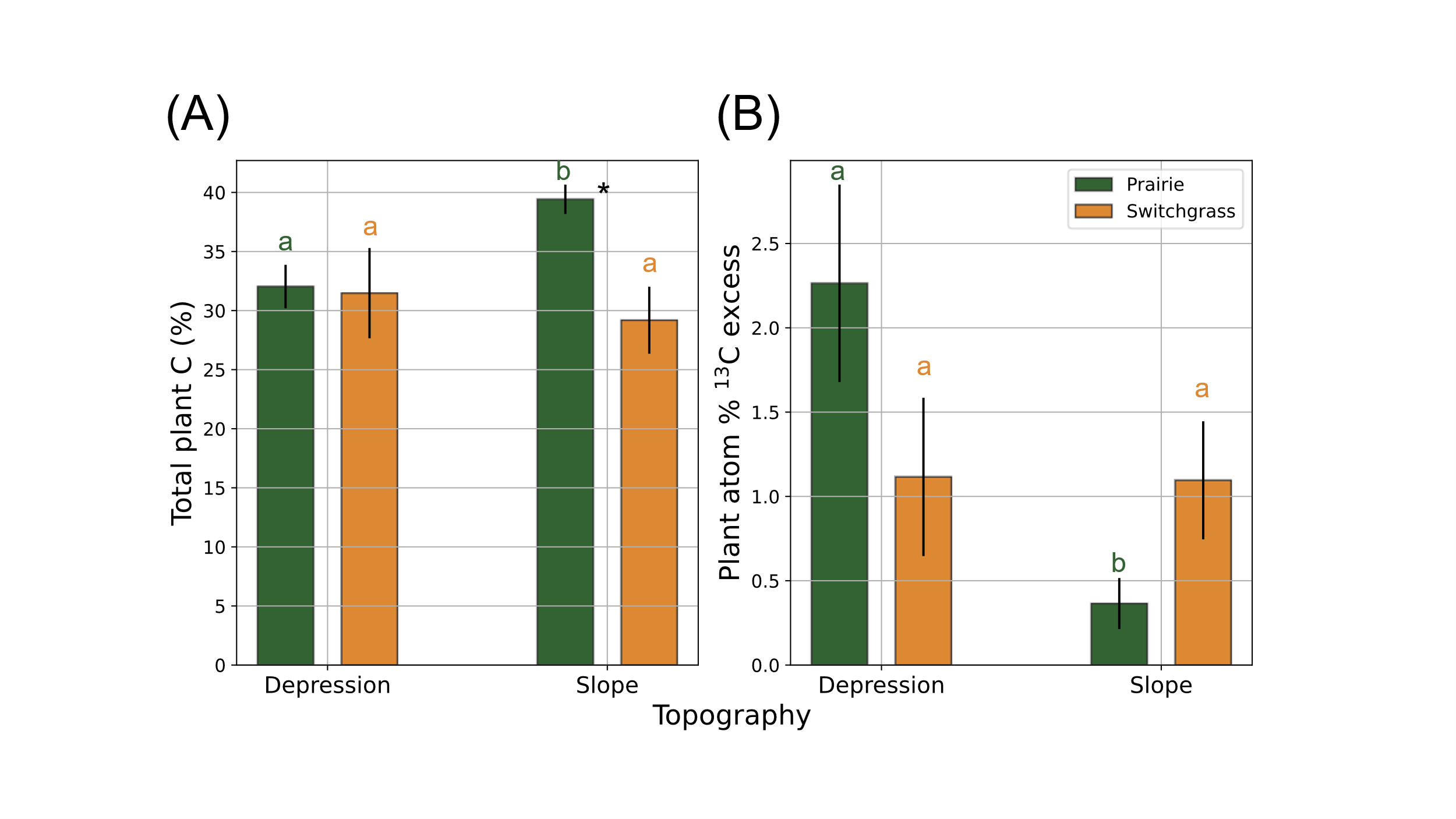


**Figure S1.** Total C (A) and atom %^13^C excess (B) of the switchgrass belowground biomass grown in the soils from multi-year monoculture switchgrass and restored prairie cropping systems in topographical slopes and depressions. Shown are means with error bars representing standard errors. Asterisk * indicates the significant difference between the two system (prairie vs. switchgrass) within the same topography and biomass type (p< 0.10). Letters mark significant differences between topographical positions within the same system and biomass type.

.

**Table S1**. ANOVA F-test results (p-values) for the plant tissue, plant system, topography, and their interaction effects for C contents in newly grown switchgrass plant. Only p-values less than 0.10 were underlined. The same statistical results were presented in Fig. 2.

| **Effect** | **Plant total C** | **Plant atom%^13^C excess** |
| --- | --- | --- |
| Tissue | < 0.01 | < 0.01 |
| System | 0.08 | 0.44 |
| System x Tissue | 0.04 | 0.00 |
| Topography | 0.35 | 0.07 |
| Topography x Tissue | 0.34 | 0.92 |
| System x Topography | 0.07 | 0.20 |
| System x Topography x Tissue | 0.12 | 0.09 |

**Table S2**. ANOVA F-test results (p-values) for the plant system, topography, plant treatment, and their interaction effects for the soil C, microbial biomass C, and cumulative CO_2_ mineralization. Only p-values less than 0.10 were underlined. The same statistical results were presented in Fig. 3, 4, and 5A.

| **Effect** | **Soil total C** | **Soil atom%^13^C excess** | **MBC** | **MB atom%^13^C excess** | **Cumulative CO_2_-C** |
| --- | --- | --- | --- | --- | --- |
| System | 0.01 | 0.25 | 0.13 | 0.04 | 0.94 |
| Topography | 0.08 | 0.22 | <0.01 | 0.49 | 0.12 |
| System x Topography | 0.10 | 0.77 | 0.10 | 0.09 | 0.43 |
| Plant treatment | 0.71 | < 0.01 | 0.07 | < 0.01 | 0.01 |
| System x Plant treatment | 0.42 | 0.22 | 0.70 | 0.13 | 0.73 |
| Topography x Plant treatment | 0.81 | 0.09 | 0.38 | 0.90 | 0.05 |
| System x Topography x Plant treatment | 0.83 | 0.82 | 0.53 | 0.23 | 0.13 |

**Table S3**. ANOVA F-test results (p-values) for the plant system, topography, plant treatment, day of incubation. and their interaction effects for ^13^C-CO_2_ mineralization. Only p-values less than 0.10 were underlined. The same statistical results were presented in Fig. 5B and C.

| **Effect** | **atom%^13^C-CO_2_ excess** |
| --- | --- |
| System | 0.50 |
| Topography | 0.10 |
| System x Topography | 0.36 |
| Plant treatment | < 0.01 |
| System x Plant treatment | 0.73 |
| Topography x Plant treatment | 0.08 |
| System x Topography x Plant treatment | 0.40 |
| Day | < 0.01 |
| System x Day | < 0.01 |
| Topography x Day | 0.00 |
| System x Topography x Day | 0.19 |
| Plant treatment x Day | < 0.01 |
| System x Plant treatment x Day | 0.00 |
| Topography x Plant treatment x Day | 0.00 |
| System x Topography x Plant treatment x Day | 0.83 |
